# New Frontiers in IVF: mtDNA and autologous germline mitochondrial energy transfer

**DOI:** 10.1186/s12958-019-0501-z

**Published:** 2019-07-12

**Authors:** Mauro Cozzolino, Diego Marin, Giovanni Sisti

**Affiliations:** 1 Department of Obstetrics, Gynecology and Reproductive Sciences, Yale School of Medicine, New Haven, CT USA; 2 Universidad Rey Juan Carlos, Madrid, Spain; 3 IVIRMA, Fundación Instituto Valenciano de Infertilidad, Avda/Fernando Abril Martorell, n° 106, Valencia, Madrid, Spain; 4 IVIRMA New Jersey, Basking Ridge, NJ 07920 USA; 5 Department of Obstetrics and Gynecology, Lincoln Medical and Mental Health Center, Bronx, New York, USA

**Keywords:** mtDNA, Autologous mitochondria, Egg precursor cells, Oocyte quality, In vitro fertilization

## Abstract

Many infertility specialists support the existence of a relationship between the levels of mitochondrial DNA and the quality of the blastocysts. Despite the extensive use of pre-implantation genetic testing for aneuploidy, a significant percentage of euploid embryos do not implant even though the endometrium is normal. Mitochondrial DNA may be used as a new test in evaluating embryonic vitality.

Ovarian aging leads to a decrease in the quantity and quality of oocytes and aged oocytes have a reduced number of mitochondria. Mitochondria are the energy factories of the cells and their lacked could leads to lower fertilization rates and poor embryonic development. Various strategies have been tested to increase the mitochondria quantity and thus improve the quality of oocytes used in in vitro fertilization. Results of ovarian rejuvenation techniques such as autologous mitochondrial transplantation have been controversial. In this review, we describe the state of the art concerning the use of mitochondrial DNA and autologous mitochondrial transplantation as new possibilities to increase success in vitro fertilization.

## Capsule

Although Autologous Germline Mitochondrial Energy Transfer seemed to be promising, a number of questions remain unsolve.

## Introduction

The identification of strategies to increase implantation, ongoing pregnancy, and live birth rates is one of the main challenges for researchers in the field of assisted reproduction. Embryo quality, endometrial receptivity and embryo transfer efficiency are key factors affecting the success of assisted reproductive treatments (ART) [1]. In the last years, investigators have developed novel technologies that reliably assess embryo viability and thus assist in the selection of embryos with the highest chance of successful transfer. However, the clinical tools for assessing embryo viability have not evolved despite substantial advances in ART, the embryologists continue to use subjective morphologic and morphometric grading systems when choosing the best embryo to transfer [2, 3]. Maternal age remains at present the main limitation in the area of ART. The peak of reproductive capacity is at about 25 years of age and decreases slightly at the age of 32, with an accelerating decline after 37 years of age [4, 5].

Aging affects both the number of oocytes that can be retrieved with the use of controlled ovarian stimulation (COS) [6] and the quality of oocytes, considering that elder women present higher rates of embryonic aneuploidy [7]. In addition, eggs of poor quality show often times lower fertilization rates or, if fertilized, they are less likely to normally develop into healthy embryos capable of completing implantation giving rise to healthy newborns [8]. Due to the relevance of aging in infertility, methods to rejuvenate and improve oocyte quality have been developed [9].

The possible reasons why poor quality oocytes fail to result in healthy pregnancies are different, ranging from meiotic anomalies causing chromosomal imbalance [10], to bioenergetics dysfunction as a result of mitochondrial alterations [11]. In the course of human life, the instability of mitochondrial DNA (mtDNA) increases with age, leading into the accumulation of mtDNA mutations resulting in the loss of mitochondrial function [12]. Evidence suggests that critical elements associated with mitochondrial biogenesis and bioenergetics likely affect embryonic capacity [13, 14]. In fact, recent data suggest that oocyte mtDNA deficiency results in poor oocyte quality and may prevent the oocyte from completing the process of fertilization [14]. Female meiosis is a highly energetic process, therefore, any deficiency in energy production within the mitochondria due to mtDNA damage can increase the risk of defective meiosis and aneuploidies [15]. In this way, in modern reproductive medicine, the research on mitochondria has become pivotal.

In this review, we discuss the role of mitochondrial DNA in In Vitro Fertilization (IVF) and the possible use of mitochondria in infertility treatments, particularly using autologous mitochondria that could potentially increase pregnancy rates. Novel strategies could provide us with both an increased number and quality of mature oocytes of women in advanced reproductive age.

### Mitochondrial DNA

**Fig. 1:**
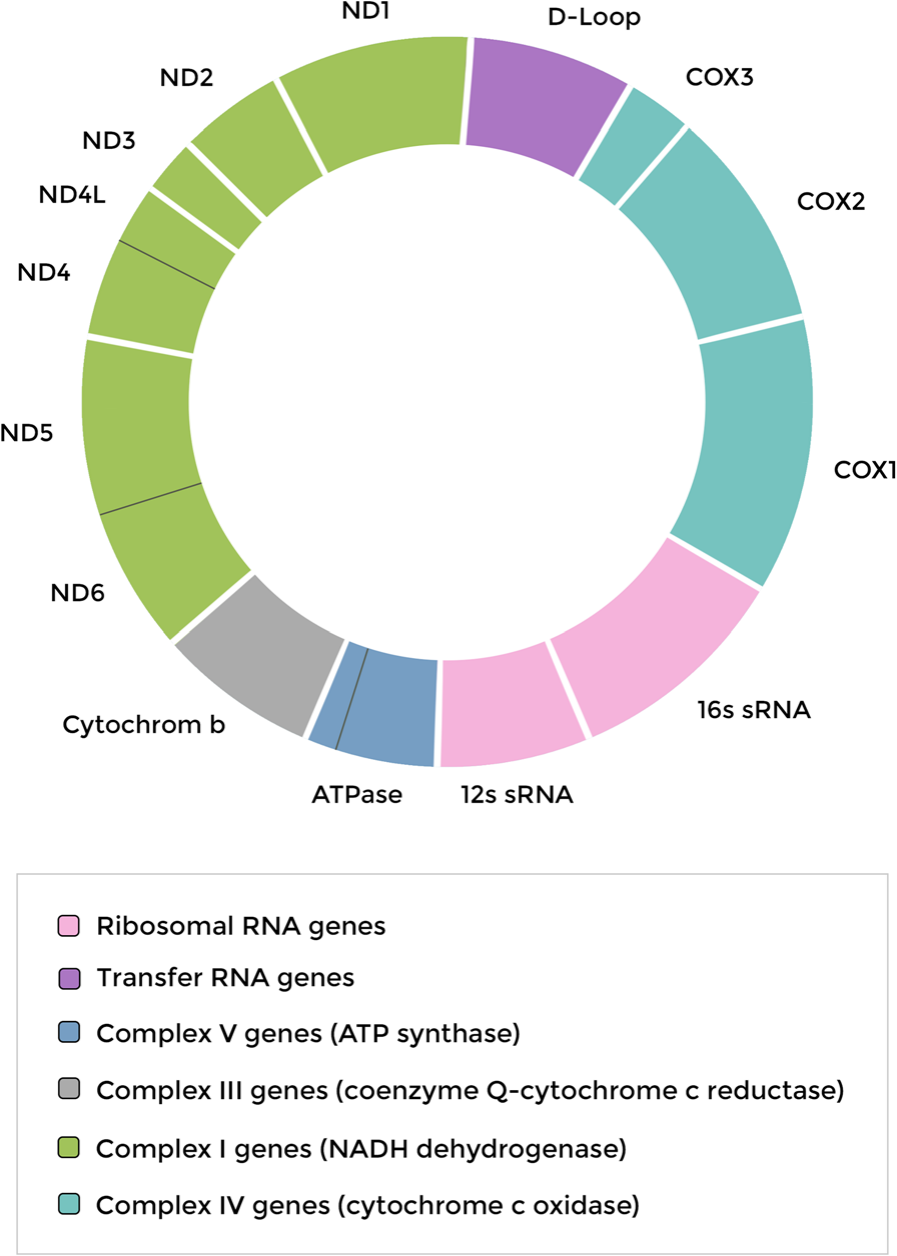
The circular mitochondrial DNA is approximately 16.6 kb and double-stranded. MtDNA contains a total of 37 genes

In the evolution of bacterial cell structures, mitochondria supposedly arose from endocytosis of prokaryotic ancestors by other cells, thus giving rise to eukartiotic symbionts [16]. Mitochondria are double membrane-bound organelles and, in a cell, can change significantly in size (0.5–10 mm) and number (1–10,000). mtDNA is a double circular DNA wire that, unlike nuclear DNA, does not contain histones or enzymes to repair DNA [17], it consists of 15 000–17 000 base pairs [18]. The heavy strand encodes 28 genes, and the light strand encodes 9 genes for a total of 37 genes. Of the 37 genes, 22 are for transfer RNA (tRNA), two are for the small and large subunits of ribosomal RNA (rRNA), and 13 are for proteins (polypeptides) [19] (Fig. 1). Thirteen proteins encoded by the mtDNA are part of the electron transport chain (ETC), which consists of five complexes localized to the inner mitochondrial membrane which are involved in the production of adenosine triphosphate (ATP) [20] through oxidative phosphorylation (OXPHOS). Only 13 of the 80 proteins encoded by mitochondrial genes are involved in the mechanisms of cellular respiration [19]. Transfer of different mitochondrial genes into the nuclear genome probably occurred in order to keep parts of the plant’s most vulnerable nuclear DNA away from energy production within the same mitochondria, where it generates a large amount of reactive oxygen species (ROS) [21]. Therefore, in order to ensure the correct functioning of the ETC and the consequent production of energy inside the cell, it is essential that the processes of transcription and translation from both nuclear and mitochondrial DNA are efficient. MtDNA is not surrounded by protective histones, unlike nuclear DNA, and is close to the ETC complex that generates large amounts of ROS. As a result, mtDNA is extremely more susceptible to mutations (approximately 5 to 10 times more than the nuclear DNA) [22]. Moreover, mtDNA does not have all the mechanisms of DNA repair [23]. Consequently, the mutations in mtDNA accumulate over time, probably jeopardize the risk of energetic disfunctioning in the cells.

### Mitochondrial DNA and oocyte quality

The inheritance of mitochondria is tightly maternal [24, 25]. Despite the fertilization, paternal mitochondria are eliminated by the oocyte through lysosomal degradation by allogeneic (non-self) organelle autophagy [26, 27]. Recently, several authors have found that sperm mtDNA remains in pre-implantation embryos. This finding suggests that sperm mtDNA is replication competent and is not actively eliminated during embryogenesis [28]. One other explanation is that the mitophagy mechanism of the oocyte is defective, and therefore unable to remove sperm mitochondria after fertilization, embryos with persisting sperm mitochondria have been shown not to be viable.

In view of these aspects, it seems that reproduction is strictly correlated to low-level heteroplasmy between different mtDNA genotypes. Heteroplasmy is the presence of more than one mtDNA variant in the cell, as opposed to homoplasmy, in which all mtDNAs in a cell are identical in sequence [29]. An acceptable level of heteroplasmy may be close to that introduced by sperm, oocytes derived from the same female show different levels of heteroplasmy. The concept of mitochondrial genetic bottleneck may explain these different levels of heteroplasmy [30], during the production of primary oocytes, a selected small number of mtDNA molecules are transferred into each oocyte. The rapid replication of mtDNA population is associated with oocyte maturation. This restriction-amplification event can lead to a random shift of mtDNA mutational load between generations [31, 32].

Mitochondria are the source of the production of cellular energy in the form of ATP, which in oocytes is essential for a successful assembly of the meiotic spindle, proper segregation of chromosomes, and maturation, fertilization and pre-implantation embryogenesis [13, 33, 34]. In addition, mitochondria are involved in several other cellular processes, including ion flows and management of the reduction-oxidation state, which are central to fertilization and development [33]. Consequently, an alteration of these mechanisms can compromise the potential development of oocytes and embryos. In fact, the data currently available on animal models and clinical results show that mitochondrial dysfunction could favour the process of aging in the ovaries. The aging process includes a reduction in the total number of mitochondria and in the potential of the mitochondrial membrane. Specifically, there could be abnormal oxidative phosphorylation status, decreased bioenergy capacity, mitochondrial aberrant localization and aggregation, sub-optimal mtDNA content, and a high mutational load of mtDNA [35–38].

The structure of mitochondria found in mature MII oocytes differs from those of somatic cells as they are small (diameter < 1 mm), spherical, and contain fewer and truncated cristae surrounding a dense matrix [39]. An important feature of mitochondrial physiology resides in the ability to amplify in number when they are in the stage of the primordial oocyte, from about 6,000 mitochondria, until mature oocyte at the end of metaphase II (MII) when the oocytes contain 300,000 to 400,000 mitochondria or more [40]. The expansion in the number of mitochondria increases the energy requirements in order to cope with fertilization and cell divisions during the embryonic development stages, up to the blastocyst stage [33, 41].

Surprisingly, in spite of sharp changes in mitochondria morphology noticed during early preimplantation embryo development, a total number of mitochondria and mtDNA copy number seem to remain unchanged during cleavage divisions, in this way the oocyte has become the primary source of mitochondria for cleavage and blastocyst stage embryos [19]. This hypothesis seems plausible because the replication of mtDNA is not reactivated in the embryo until the blastocyst stage [42]. Therefore, a dilution of the number of mitochondria is observed as the embryo expands. This idea is supported by a reduced mtDNA content of blastomeres in blastocyst stage embryonic cells compared to oocyte cells [13]. Older patients with infertility have an abnormal mitochondrial activity in the oocytes and reduced production which limits normal embryonic development. The scarce dowry of mitochondria could lead to a faulty embryonic development [38, 43] and the integration of mitochondria could improve fertilization [43]. The functional status of mitochondria contributes to oocyte quality [38]: higher-quality oocytes, assessed by morphology, contain significantly higher ATP levels and are more likely to move forward to the blastocyst stage after fertilization [34]. Indeed, inappropriate mitochondrial activity at the pronuclear stage is associated with early developmental arrest and demise [44].

### Mitochondrial DNA as a marker of in vitro fertilization

In recent years, there has been a growing interest in the evaluation of mtDNA as a possible potential biomarker of embryo vitality. Each mitochondrion exhibits 1–15 mtDNA copies in somatic cells [17], whereas germline cells, such as oocytes, contain only one copy per organelle [32, 45]. In the oocyte, mtDNA increases considerably until the stage immediately preceding fertilization, which coincides with ovulation. During embryogenesis, mtDNA is equally distributed in the cells of healthy embryos. The total amount of mtDNA is divided between the cells of the embryo at each mitotic division, and, therefore, on day 5–6 of development, the cells of the embryo contain a significantly lower quantity of mtDNA compared to that of the original oocyte/zygote, thus showing that the replication of mtDNA does not resume before blastulation. Considering that the number of mitochondria and the content of mtDNA in oocytes correlate with the outcome of fertilization and embryo quality [13, 38, 46, 47], some authors have suggested a minimum threshold for the number of mtDNA copies within the MII oocyte to enable embryonic development after fertilization [43, 48].

Recent studies have proposed to quantify mtDNA in the granulosa cumulus cell and embryo trophectoderm to predict embryo quality and viability [49–52]. Boucret et al. (2015) have shown that the amount of mtDNA in cumulus cells is comparable to the amount detected within the oocyte for each cumulus-oocyte complex [53]. With that in mind, they hypothesized that mitochondria can be used as markers of oocyte competence. Dumesic et al. (2016) demonstrated a positive correlation between the ability of mitochondrial membrane potential of cumulus cells to withstand stress and the number of mature oocytes collected [54]. In the oocytes from women with ovarian failure, some authors have shown fewer copies of mtDNA, assuming a possible change of mitochondrial quantity, function and DNA integrity during senescence of human oocytes [35, 36, 51]. From the beginning, there was evidence to support a negative correlation between concentration of mtDNA in human MII oocytes and age [55]. It has been hypothesized that the decrease in the vitality of the oocytes, with age, can be explained at least in part with a reduced amount of mtDNA together with an increase in the mutation rate [55]. Subsequent publications have reported a clear reduction in human oocytes in mtDNA levels over the course of reproductive senescence [37], and a similar trend was revealed in a biopsy of polar body [56]. Conversely, a few studies have failed to find any association between the amount of oocyte mtDNA and the aging process [38, 46, 57].

In patients with diminished ovarian reserve (DOR) the levels of mtDNA in oocytes were lower than women with a normal ovarian reserve [35, 36, 53]. These data strongly suggest that in the MII oocytes a high mtDNA copy number is needed in order to support fertilization and early development embryo up to the resumption of mtDNA replication in the blastocyst phase. The mtDNA content in embryos represents the amount of mtDNA within the oocytes before fertilization because mtDNA does not replicate until the blastocyst stage [45]. In other words, the amount of mtDNA in the blastocyst is the result of the progressive dilution of the mtDNA of the fertilized oocyte, through the cellular divisions of the embryo [58].

The mtDNA copy number in the blastocyst phase in elderly women is higher [51]. The aneuploid blastocyst occurs with mitochondrial copies higher than with the euploid blastocyst, while the same has been observed with a euploid blastocyst that cannot implant compared to those that implant [50, 51].

Fragouli et al., have highlighted some key aspects in their results. They found a higher number of mtDNA copies in older women, in aneuploid embryos and in the euploid blastocyst with implant failure. They calculated a threshold mtDNA value above which pregnancy did not implant [51]. Diez-Juan et al. confirmed similar results and proposed a similar mtDNA score to predict implantation potential/embryo viability [50]. Unfortunately, neither of these two studies takes into account the distribution of mtDNA copy number among the same woman’s embryo cohort, nor tests whether an embryo from the same patient is more likely to be implanted if it has a higher mtDNA copy number. These studies have recently been called into question by Victor et al. (2017), which did not find any significant difference between mtDNA blastocyst scores with regard to embryonic vitality, ploidy or maternal age [59]. On the other hand, Victor et al.’s study is affected by several methodologic and technical issues [15, 60].

In a retrospective study on 1505 blastocysts, Ravichandran et al. proposed mtDNA quantification as a biomarker for the identification of a subset of blastocysts that are non-viable despite being chromosomally normal [60]. The authors concluded that increased aneuploidy rates and poorer blastocyst quality were associated with higher mtDNA [61].

Treff et al. in 2017 concluded the largest study to date evaluating the predictive value of mtDNA content using sibling blastocysts of discordant sex, showing that relative mtDNA levels did not distinguish between embryos that implanted and those that failed to the implant [62]. This design is the only one allowing for control of patient-specific variables in reproductive medicine. Based on these results, the currently available data suggest that mtDNA quantification needs further study before its clinical use to augment selection in addition to more validated quantification techniques [62]. Age was inversely correlated with the mtDNA load, which is inconsistent with the previous studies [51, 60], whereas a better embryo grade was correlated with a lower mtDNA copy number. In a retrospective study on 465 consecutive preimplantation genetic screening (PGS) cycles of 402 women undergoing preimplantation genetic testing, the authors identified embryos with superior morphology and smaller mtDNA quantities [61]. A recent large retrospective study investigated the possibility of a relationship between mtDNA content and blastocyst quality [63]. Interestingly, although mtDNA levels were found to be predictive of very-poor or very-good quality embryos in the whole sample, the results were not statistically significant when examining euploid blastocysts only.

One blinded prospective non-selection study analyzed 199 euploid blastocysts: 57 did not implant and nine of those exhibited abnormally high levels of mtDNA. A direct association between mtDNA and the implantation potential is probably too optimistic, the mitochondrial evaluation seems to better predict the non-vitality of an embryo rather than facilitate the selection of the best embryo to be transferred. All information about the potential use of mtDNA in embryos or blastocyst to predict IVF outcomes is summarized in Table 1.

**Table 1:** Investigation of mtDNA in human preimplantation embryos/blastocysts which had successfully reached the blastocyst stage of development:correlations between the amount of mtDNA and implantation rate, aneuploidy rate, and morphology of the embryo. The studies were heterogeneous: each study used specific selected samples, statistical tests and sub analysis of results

	Study design	Number total embryos and blastocysts analysed	Differences in mtDNA quantity in implanted vs non implanted pregnancies	Differences in mtDNA quantity in euploid vs aneuploid pregnancies	Differences in mtDNA quantity in poor vs good embryo morphology
Fraguoli et al., 2015 [14]	OP	379	lower amount of mtDNA (*p* = 0.007)	lower amounts of mtDNA (*p* = 0.006)	N/A
Diez-Juan et al., 2015 [50]	OR	27027	lower amount of mtDNA (*p* < 0.02)	N/A	not statistically significant
Victor et al., 2017 [59]	OR	1396	not statistically significant	not statistically significant	N/A
Ravichandran et al., 2017 [60]	OR	1505	lower amount of mtDNA (*p* = 0.030)	N/A	N/A
Fraguoli et al., 2017	OP	199	lower/normal amount of mtDNA (*p* < 0.0001)	N/A	N/A
Treff et al., 2017 [62]	OR	374	not statistically significant	N/A	N/A
Klimczak et al., 2018 [63]	OR	1510	not statistically significant		higher amount of mtDNA (*P* = 0.003)
De los Santos et al., 2018 [61]	OR	465	N/A	lower amount of mtDNA (*p* < 0.001)	higher amount of mtDNA (*p* < 0.03) *

*OR* observational retrospective, *OP* observational prospective

*in euploid embryos

### Heterologous sources of mitochondria: partial cytoplasm transfer, total cytoplasm transfer, spindle transfer

**Fig. 2:**
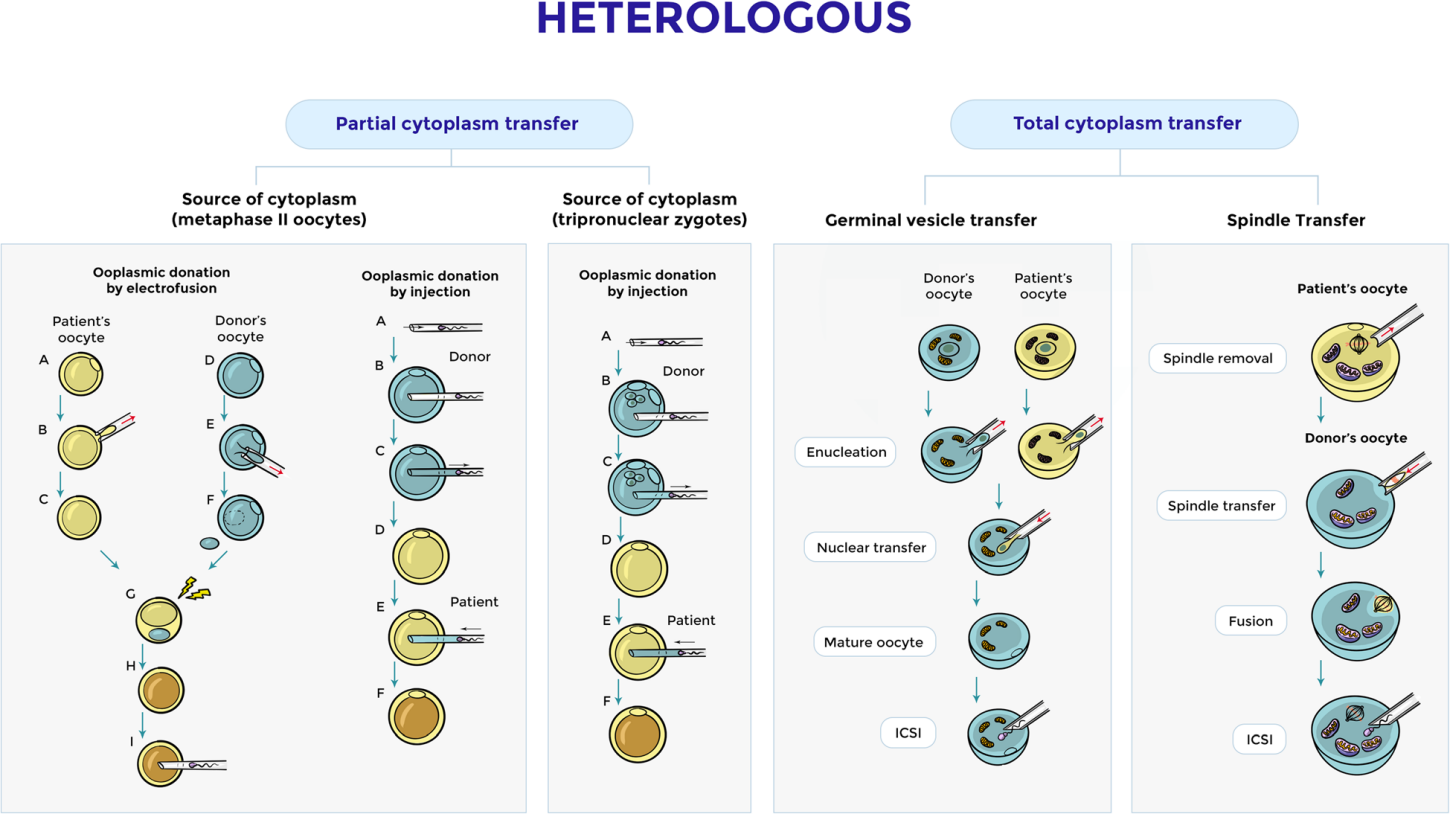
Heterologous sources of mitochondria: partial cytoplasm transfer, total cytoplasm transfer of germinal vesicle and spindle transfer

Early first experiences had shown that the ooplasm transfer of healthy oocytes was able to block stopping embryos obtained from abnormal oocytes [64]. In the past, research made it possible transferring the cytoplasm of a donor into oocytes prone to abnormal development in order to obtain good quality embryos [65]. Patient candidates for this type of technique were mainly those in advanced age, poor responders, with repeated failures of IVF. This technique managed to lead to the birth of healthy babies. The success of this procedure was due to the mitochondrial transfer of the ooplasm of the donor to the receiving oocyte. The mitochondrial replacement includes heterologous or autologous, the first may concern to a partial or total transfer of the cytoplasm. In the case of partial cytoplasm transfer, two approaches were initially investigated: fusion of donor oocyte cytoplasm (membrane-enclosed cytoplasm fraction) with the patient oocyte, or direct injection of a small amount of donor cytoplasm into a patient oocyte [65, 66] (Fig. 2).

Total cytoplasm transfer refers to the thorough replacement of a pathologic cytoplasm with a competent one with the use of nuclear transfer technology [67]. Briefly, spindle transfer consists of transference of the birefringent spindle, enclosed into a karyoplast, from a patient oocyte to the perivitelline space of a cytoplast (MII enucleated donor oocyte) containing healthy mitochondria [68] (Fig. 2).

Membranes from the karyoplast and cytoplast should be integrated before fertilization of the generated oocyte. In 2017 Zhang et al. reported the first birth of a healthy child derived from IVF using a successfully reconstituted human oocyte from a female carrier of mitochondrial disease, a 36-year-old woman with asymptomatic Leigh syndrome with a history of two deceased children from a mutation in mtDNA 8993 T > G in subunit 6 of the ATPase gene. This new birth strongly suggests that spindle transfer can significantly reduce the load of mutated mtDNA [68]. Despite the numerous and encouraging successes of this technique, it aroused a great deal of criticism for the possibility that the ooplasm transfer resulted in offspring carrying mitochondria from both the donor and the recipient, thus creating mitochondrial heteroplasmy [69]. The immediate effect of mitochondrial heteroplasmy is currently unknown and could have potentially deleterious late physiologic consequences [70, 71]. With this technique, it is almost inevitable that a certain amount of cytoplasm is transferred into the donor’s enucleated oocyte in order to maintain nuclear integrity, thus introducing mtDNA along with the nucleus, which leads to heteroplasmy in the reconstituted oocyte. This spindle transfer technique accomplished a less than 6% carryover rate. Several controversies have been raised, some relating to ethical concerns [72–76] and others to the weakness of the technical aspects of the procedure [77].

In the study by Zhang et al., the electrofusion procedure was used to archive membrane fusion to avoid virus protein transmission [68], inducing oocyte activation. The electrofusion procedure, as suggested by Tesarik et al., was responsible for an increased rate of aneuploidy. A recent study [78] using similar methodology seems to overcome the increased aneuploidy rate to restore embryo developmental potential in mouse oocytes because the resulting offspring showed the health and behavioral status similar to control offspring. Probably, the quality of the recipient oocyte is the responsible for the impairment of resulting embryos rather than the fusion method [79].

The spindle transfer could be used also in the case of cleavage state arrest. The genes of subcortical maternal complex (SCMC) play important roles during embryonic development and using whole-exome sequencing novel biallelic mutations in the SCMC genes TLE6, PADI6 and KHDC3L were identified in patients with embryonic developmental arrest. A mutation in TLE6 was found in a patient with cleaved embryos that arrested on day 3 and failed to form blastocysts. PADI6 and KHDC3L are mutated in embryos that arrested at the cleavage stage and morula stage. These findings provide further evidence for the important roles of TLE6, PADI6, and KHDC3L in embryonic development [80]. Recently, authors proposed that not only mitochondrial diseases but a rare condition that can lead to an embryo arrest named preimplantation embryonic lethality could possibly be overcome by pronuclear transplantation [81]. In young women the cleavage arrest could be resolved with spindle transfer, in two cases the all mitochondrial variants of the donor were detected in the fetus and in the other one, the majority of mitochondrial variants detected in the child are shared with the mitochondrial donor and not with biological mother [81].

### Autologous sources of mitochondria: Oogonial stem cells (OSCs) and development of AUGMENT procedure

The benefits of this mitochondrial enrichment are clearly evident during the early developmental stage. In 2004 Johnson et al., reported the existence of mammalian female OSCs assuming an inherent capacity of the ovaries to generate new oocyte-containing follicles in a regulated fashion [82]. Thus, calling into question the long-standing belief that female mammals were born with an oocyte heritage that was not renewable.

Although this change of thinking was initially encountered with resistance due to the possibility of defeating widely accepted theories [83], subsequent studies have since independently shown that mouse OSCs can be isolated from adult ovaries for long-term propagation in vitro [84, 85] and for the generation of fertility-competent oocyte in vivo following intraovarian transplantation into female mice receiving chemotherapy [84]. Johnson et al. showed the expression of germline markers in bone marrow-derived cells. Furthermore, bone marrow and peripheral blood transplantations restored the oocyte production in wild-type mice sterilized by chemotherapy [86].

OSCs clearly demonstrated their ability to generate competent oocytes for fertilization, giving rise to a viable offspring [87–89]. Guo et al*,* supported the presence of active germ stem cells in postnatal ovaries and their function in replenishing primordial follicle pool under physiological conditions [90].

Like their murine counterparts, human OSCs possess a unique gene expression profile consistent with a primitive germ cell identity, and these cells can be established in culture for ex vivo propagation and in vitro oocyte formation studies [88]. The authors evidenced that human OSCs can directly support new oocyte formation and that adult human ovarian tissue remains amenable to de novo *follicle* formation. The identification of a population of adult germ stem cells, entirely dedicated to the production of new oocytes in the ovaries of women in reproductive age, has offered an unprecedented opportunity to test a completely new set of fertility enhancement technologies [71].

**Fig. 3:**
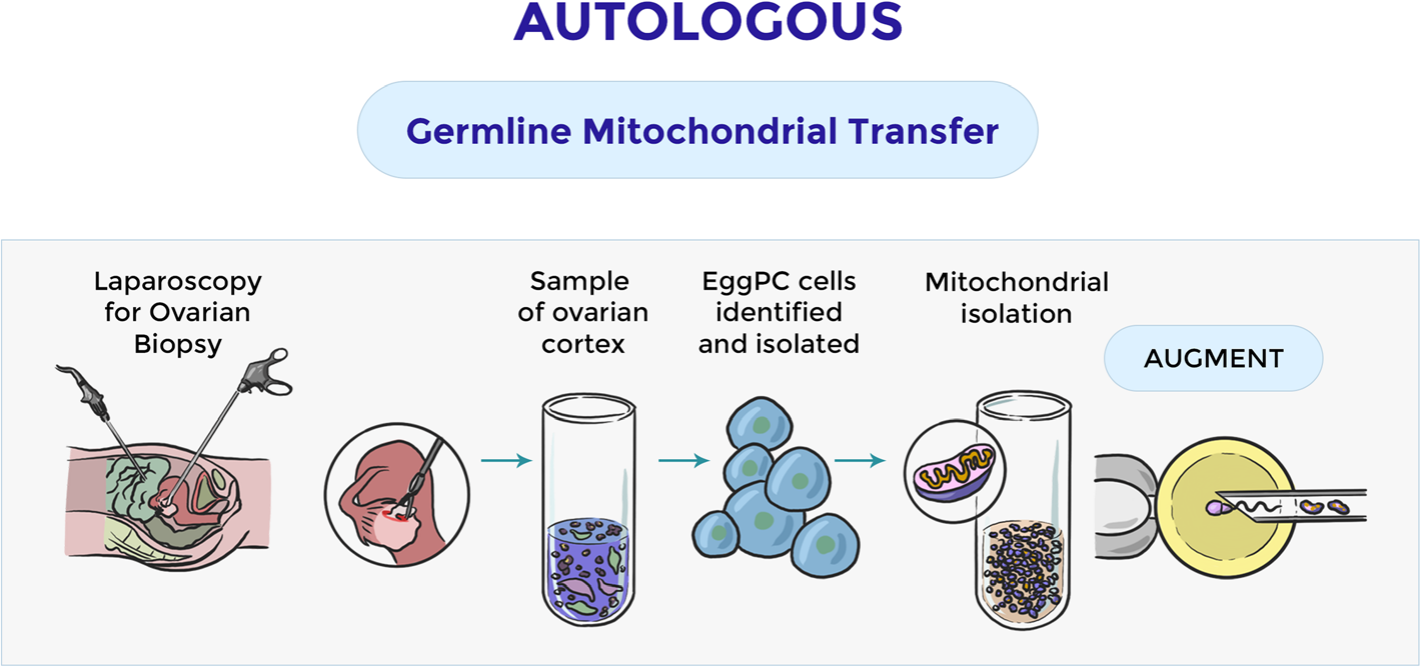
Autologous source of mitochondria: the Augment procedure

The simple fact that OSCs are natural endogenous precursors of oocytes underlines a very important and clinically useful feature of these newly discovered cells: The mitochondria present in OSCs are the same as those found in oocytes, here is where the first type of cell differs from the second [84, 88]. For this reason, the administration of OSC-derived mitochondria from an IVF patient, together with sperm from ICSI, would provide an autologous means of recapitulating the benefits of heterologous cytoplasmic transfer without the disadvantage of having “foreign” mitochondria present in the resulting embryos and offspring. The idea of being able to improve the quality of oocytes through the infusion of mitochondria generated by an autologous source of oocyte precursor cells with great energy potential has given rise to the concept of autologous germline mitochondrial energy transfer (AUGMENT) (Fig. 3). As mentioned above, OSCs and oocytes, as a cellular continuum, possess the same mitochondrial population. In the mitochondria of OSCs, mtDNA is located in the immediate vicinity of the transport chain of mitochondrial electrons, responsible for ATP generation, as results of these bioenergetics process, the cells produce reactive species of oxygen (ROS). This exposure to ROS, combined with the circular nature of the mtDNA structure without a histonic spine, and the absence of effective repair mechanisms of mtDNA in the cells, results in a high susceptibility of mtDNA to mutational damage that accumulates with age [80, 84]. A comparison of the mutation rate of mtDNA in human OSCs with somatic cells from the same ovarian tissue showed that OSCs cells have a much lower mutation rate than somatic cells [81, 85]. Probably these cells, with low metabolic activity, as they are dormant cells, have a lower rate of DNA damage due to age than the counterpart of ovarian somatic cells [81, 85]. In addition, direct comparative analysis of the in vitro ATP formation capacity of a fixed amount of mitochondria isolated from human OSCs; human embryonic stem cells; human embryonic somatic stem cells; pluripotent stem cells of human origin, or mesenchymal stem cells derived from human bone marrow; found OSCs to be the greasy ones that could give the greatest bioenergetics boost [81, 85]. However, at present, there are still important restrictions that need to be taken into account. The first one is the source of the ovarian cortical tissue to be used to “house” the purified OSCs for differentiation. Clinically, the use of autologous tissue would be optimal, but, with advancing age, the intraovarian environment may become inadequate to OSCs differentiation [91]. One of the possibilities on which some laboratories work is to use undifferentiated or pre-granular cells that aggregated with OSCs and other ovarian somatic cells could reconstitute autologous ovarian tissue ex vivo [92]. The second problem concerns the evaluation of the competence of eggs produced by OSCs. Human eggs produced by OSCs can be compared with endogenous eggs for various endpoints, such as metabolic and parthenogenetic profiles potential, bovine eggs produced by stem cell technologies could be fully assessed by means of fertilization, embryo development and live births [93].

Surely, there is still a long way, but we must not forget that about two decades ago, postnatal oogenesis and the reconstitution of the entire life cycle of the cells female germline in vitro in mammals were science fiction. Time will tell if the current science about OCSs will blur in clinical practice tomorrow.

### Autologous germline mitochondrial energy transfer: human experience

Mitochondria isolated from a woman’s own egg OSCs appear to be similar to young oocyte mitochondria and can serve as an autologous source of mitochondria to be injected into deficient oocytes, thereby avoiding the concerns raised with donor oocyte mitochondrial injection [94]. These autologous mitochondria were injected at the time of ICSI into the patient’s own oocytes. The autologous mitochondrial treatment of mitochondrial injection is based on previous reports related to cytoplasmic injections of oocytes from a donor [57], those numerous studies based on animals have shown that the addition of mitochondria during in vitro fertilization and treatment can improve the quality of the embryo [43, 95].

Since 2015, three international fertility centers have successfully developed and launched AUGMENT as a therapeutic option for women with a history of consistently poor assisted reproduction outcomes. Two of these sites, the Toronto Centre for Assisted Reproductive Technologies (TCART) Fertility Partners (Toronto, Canada) and FAKIH IVF (Dubai, United Arab Emirates) have recently reported their first clinical experiences with 93 patients treated with AUGMENT who agreed to participate in the OvaScience Global Registry program [87]. Subsequently, the third site from Europe reported the results of the other 11 women who received AUGMENT in Gen-Art IVF treatment [96]. At all the three centers, 104 patients with 369 previous IVF cycles were recruited [87, 96]. Pregnancy rates obtained by patients in previous IVF cycles were 5.2 and 1.3%, respectively. In the same patient population, only a single cycle of AUGMENT produced a clinical pregnancy rate of 35% in TCART and 22% in FAKIH IVF, with an ongoing pregnancy rate and a live birth rate of 26 and 18% per cycle initiated [87, 96].

In evaluating the collective data of the two sites with the largest clinical experience with AUGMENT (*n* = 93 patients), it is clear that AUGMENT increases clinical pregnancy rates per cycle initiated, from 3 times (TCART) to 6 times (FAKIH IVF). These are prospective cohort [40] or descriptive studies with small numbers of patients [96], carrying low quality of evidences. In the study by Fakih et al. [40], there was a difference in the allocation of eggs to the AUGMENT compared to IVF alone (171 vs. 106, respectively), which does not reflect a methodologically correct division of patients. Fakih et al. reported a marked improvement in pregnancy rates above the historical IVF success rate for these latter patients. However, the scientific community did not very much agree on the quality of the results obtained, stating that these results could have been biased by the inter-cycle variability of IVF [40]. Oktay et al. reported results from 10 patients from Genart Ankara (Turkey) with at least two IVF failures due to bad oocyte/embryo quality that completed the Augment technique and had embryo transfer [96]. In the same way as in the other two studies, this one is limited by protocol and study population because not all patients followed the same procedures.

The subsequent study by Labarta et al. in 2018 clarified some fundamental aspects, and the methodological rigor of the study leaves no room for negative interpretation of the quality of their results [97]. In comparison with the two previous clinical studies, the strength of this study is that an intrapatient and an intracycle comparison were performed, through the allocation of sibling oocytes to receive either AUGMENT or conventional ICSI, thus avoiding bias regarding other factors that can affect the quality of the oocytes even in the same patient (inter-cycle variation) [97]. In addition, the study was carried out in triple blind, where no one between the medical patient and embryologist knew what treatment was being used, selecting the best embryo for transfer. The results of Labarta et al. were better in terms of pregnancies than those previously published by other groups to test the AUGMENT technique, obtaining a live birth rate of 41.2% compared to Oktay et al.*,* who obtained a 9% ongoing pregnancy rate in 11 patients [96], and Fakih et al. [87], who published the results of two groups with an ongoing pregnancy rate of 18% in a sample of 59 patients and 26% in another of 34 patients.

In the study by Labarta et al., the pregnancy result was comparable in the two groups: the cumulative ongoing pregnancy and live birth rate per transferred embryo was 41.2% in the AUGMENT group and 41.7% in the Control group. This result demonstrates that AUGMENT was not able to improve the reproductive prognosis, at least in patients with previous IVF failure. In the cohort by Labarta et al.*,* the embryo morphology, the morphokinetic variables, euploid status, or mtDNA content were similar between the AUGMENT and control group [97]. An interim analysis of the study by Labarta et al. led to the premature discontinuation of the study before the required number of patients was obtained to reach the desired clout [97]. Unfortunately, the authors do not consider a very plausible biological explanation of why Augment does not work. It was generally assumed that aneuploidy has its main origin from errors in female meiotic divisions [98], the new mitochondria were injected into MII oocytes, but the vast majority of aneuploidies occur during MI [99], so actually, they injected too late.

## Conclusion

MtDNA is an important source of information about the energy for the oocyte intended to be artificially fertilized by the male gamete. Definitely, optimistic predictions for the potential use of mtDNA quantification to anticipate embryo viability. The use of mitochondrial DNA have slowly been replaced by a serious perplexity and the researchers are focusing on a better understanding of the clinical implications. Mitochondrial medicine is more complex than we believe, and it is not possible to reduce it to the expression of a simple biomarker, in the same way, embryo capacity is linked to multiple factors, making the task even more complex. MtDNA still remains an exciting field of developments in IVF and more research is needed to unravel new benefits for our patients.

Various strategies have been tested to increase the mitochondria content and thus improve the quality of oocytes. It seems plausible that the transfer of autologous mitochondria would add energy and improve IVF outcomes. Also, it could solve the ethical objections associated with heteroplasmy without the hypothetic risk of mitochondrial disease inheritance. Unfortunately, this intuitive idea has only been weakly supported by one study with important methodological flaws and has not been confirmed by the only randomized controlled trial available. Based on current evidence, we do not support the use of autologous mitochondrial injection as an ovarian rejuvenation technique in patients with multiple previous IVF failures. Now there are many alternative and innovative approaches, unfortunately still all in very early stages, so the search for ovarian rejuvenation must necessarily continue, we are still far from solving this great challenge.

## Data Availability

Not applicable.
